# Impacts of Square Stepping Exercise on Physical-Cognitive Function, Biomarkers, Body Composition and Mental Health in Healthy Senior Aged 60 and Above: A Systematic Review

**DOI:** 10.3390/healthcare12232325

**Published:** 2024-11-21

**Authors:** Juan Manuel Franco-García, Jorge Carlos-Vivas, Antonio Castillo-Paredes, Noelia Mayordomo-Pinilla, Jorge Rojo-Ramos, Jorge Pérez-Gómez

**Affiliations:** 1 Health, Economy, Motricity and Education (HEME) Research Group, Faculty of Sport Sciences, University of Extremadura, 10003 Cáceres, Spain; jmfrancog@unex.es (J.M.F.-G.); jorgepg100@unex.es (J.P.-G.); 2 Physical Activity for Education, Performance and Health (PAEPH) Research Group, Faculty of Sport Sciences, University of Extremadura, 10003 Cáceres, Spain; jorgecv@unex.es; 3 Grupo AFySE, Investigación en Actividad Física y Salud Escolar, Escuela de Pedagogía en Educación Física, Facultad de Educación, Universidad de Las Américas, Santiago 8370040, Chile; 4 BioErgon Research Group, Faculty of Sports Sciences, University of Extremadura, 10003 Cáceres, Spain; nmayordo@alumnos.unex.es (N.M.-P.); jorgerr@unex.es (J.R.-R.)

**Keywords:** body composition, executive function, fear of falling, muscle strength, physical fitness

## Abstract

**Background**: The aim of this systematic review is to analyze the effects of Square Stepping Exercise (SSE) on physical and cognitive function in older people, including its effects on biomarkers, body composition and mental health, focusing only on research that assessed the efficacy of SSE-based interventions. **Methods**: PubMed, Web of Science, Scopus and Cochrane databases were searched from June 2006 to June 2024 according to the PRISMA guidelines. The main search terms used were related to “older people” and “square-stepping exercise”. Controlled trials that included at least one intervention group focused on SSE were included. Participants had to be healthy, without physical or cognitive impairment, and the studies published in English or Spanish. The methodological quality of the selected research was assessed using the Physiotherapy Evidence Database (PEDro). **Results**: Twelve studies were selected from a total of 444 original records, with a total sample size of 577 participants. The health parameters of the participants were homogeneous, with ages ranging from 60 to 80 years. Significant gains were reported in certain physical function assessments, including balance, lower body strength and power, gait speed and flexibility. There were also significant findings in cognitive function, particularly in general cognitive status, focused attention, response time, basic task performance, and executive function. In addition, SSE can improve metrics such as body composition, brain-derived neurotrophic factor (BDNF), and mental health characteristics. **Conclusions**: SSE has the potential to significantly improve physical function, cognitive performance and body composition, as well as provide mental health benefits and have variable effects on biomarkers and cardiovascular health.

## 1. Introduction

As life progresses, it is inevitable that physical changes caused by ageing will occur. The effects of ageing are associated with structural and functional changes that affect the sensory, neuromuscular, and cognitive systems, weakening abilities acquired throughout life [1]. Sociodemographic studies of the rate of ageing of the world’s population have revealed an alarming rate that poses an economic challenge to institutions, as the proportion of people aged over 60 is predicted to double from 11% today to 22% by 2050 [2]. Although ageing does not always affect the health of older people, it is associated with a decline in muscle strength, agility, balance and physical fitness, sometimes accompanied by a decline in cognitive function, which makes walking more difficult and increases the likelihood of falls [3,4]. Maintaining physical fitness in older people is therefore crucial.

Ageing causes a loss of muscle mass, which has a direct impact on the strength exerted by musculoskeletal tissues [5], thus affecting the risk of falls and other contributing factors [6,7]. In addition, some studies have identified strength and power as measures to prevent falls, with lower power in the sit-to-stand test and lower strength in the handgrip test in older people [8,9,10]. To maintain functional independence in older people, it is important to prevent loss of balance during the ageing process [11]. Loss of balance significantly increases the risk of falling, which has a significant impact on health and quality of life by limiting the functionality of the adult [12]. Loss of balance increases the morbidity of falls, leading to muscular and skeletal injuries and compromising gait safety [13]. Falls in older adults affect approximately 30% of people over the age of 65 each year [14]. The consequences can be serious, including fractures, particularly of the hip, leading to hospitalization, dependency and high mortality [15]. In addition, fear of falling again can limit physical activity and increase the risk of further falls [16].

Physical activity (PA) has been identified as a key factor in preventing falls and maintaining the health and quality of life of older people [17,18]. Regular physical activity improves physical abilities (muscular strength, balance, coordination, flexibility and cardiorespiratory capacity) and cognitive abilities (increased attention span, reflexes, etc.) and thus has a direct impact on the risk of falls [19,20,21]. Similarly, research has shown that PA plays a role in modulating inflammatory biomarkers and improving body composition in older adults [22,23]. Regular PA is associated with significant reductions in levels of C-reactive protein and other pro-inflammatory cytokines, such as IL-6 and TNF-α, which may reduce the risk of chronic disease and improve quality of life in this population [24]. It is also associated with increases in other markers of cognitive health and neural plasticity, such as BDNF [25], and other markers that regulate glucose metabolism, such as adiponectin and HOMA-IR [26]. In addition, PA helps to reduce fat mass and maintain or even increase lean mass, preventing conditions such as sarcopenia and sarcopenic obesity, both of which are associated with increased frailty and risk of falls in older people [27].

Active ageing, defined as PA in the later stages of life, has been shown to reduce the risk of falls. Participating in a PA program can reduce fall incidence by up to 50% by increasing overall strength, particularly in the lower limbs [28,29]. Such interventions focus on muscle strength, balance and coordination [30], but additional programs focus on gait and cardiorespiratory fitness [31,32], often with a cognitive component. These interventions have been shown to reduce the risk of falls by detecting changes in gait [33,34]. Active ageing faces a number of barriers to PA, such as the fact that many older adults do not engage in PA due to a lack of time, lack motivation, or even suffer from various complaints [35,36]; however, other studies have identified facilitators to practice, such as perceived health benefits, enjoyment, or perceived social support [37,38].

Considering the barriers and facilitators to physical activity discussed above, specific strategies have been developed to promote active ageing. In this context, the Square Step Exercise (SSE) has been developed as a low-cost and simple training protocol aimed primarily at improving lower body strength, agility, and cognitive and functional abilities [39], making it an effective training plan for reducing the risk of falls in older people [40,41]. It involves performing steps and movements in all possible directions on a thin carpet divided into different squares, under the supervision of the trainer in charge, and varying the intensity of the training according to the individual’s unique qualities [42]. Benefits are obtained at a cognitive level, as it requires attention and spatial awareness to perform the movements efficiently and safely; and at a motor level, as in these movements the trainer can instruct users to shift their weight onto the tips or heels of their feet, challenging static and dynamic balance and agility, as well as muscle strength [43,44,45], with additional improvements at the level of social interaction, as it tends to be performed in a group [46].

Despite the benefits of SSE reported in the scientific literature, no systematic reviews have been conducted to examine the effects of SSE interventions on the physical-cognitive components of healthy older people. In this sense, the hypothesis of our systematic review is that SSE has a significant impact on physical and cognitive function in older people, as well as on body composition, biomarkers associated with healthy ageing and mental health. The implementation of SSE is expected not only to improve balance and coordination, but also to reduce risk factors associated with falls and to promote the maintenance of executive function, all of which will promote a better quality of life in this population. Therefore, the aim of this systematic review is to analyze the effects of Square Stepping Exercise on physical and cognitive function in older people. We examined the effects of the treatments on biomarkers, body composition and mental health, as well as their effects on cognitive and physical function. Only trials that examined the effectiveness of therapies based on the use of the SSE were included to ensure the accuracy and relevance of the results.

## 2. Materials and Methods

This systematic review adhered to the Preferred Reporting Items for Systematic Reviews and Meta-Analyses (PRISMA) standards [47]. This review was registered in PROSPERO (CRD42024552309).

### 2.1. Literature Search Strategy

A systematic review was conducted using four internet databases: PubMed, Web of Science (WOS), Scopus, and Cochrane, from June 2006 to June 2024. The population, intervention, comparison, and outcome (PICO) strategy was used to structure the formulation of clinical and research questions and to guide the search and analysis of relevant references [48]. Only search terms related to the population (older people) and the intervention (square-stepping exercise) were included. As a result, the following search phrases, Boolean operators, and combinations were used: (“square-stepping exercise” OR “square stepping exercise” OR “square step exercise”) AND (elderly OR “older people” OR “older adult*” OR “older person*”). The reference lists of the included trials and related systematic reviews were also searched for potential eligible trials.

### 2.2. Inclusion and Exclusion Criteria

Intervention research eligible for the review met the following inclusion criteria: (1) Participants: people over 60 years old with no physical or cognitive pathologies; (2) Intervention: including at least one group which performed SSE or SSE combined with other exercise; (3) Outcomes: showing effects on physical or cognitive fitness; (4) Study design: randomized or non-randomized clinical studies; and (5) Language: studies written in English or Spanish.

Both randomized clinical trials (RCT) and non-randomized clinical trials (NRCT) were included in this systematic review. However, the results of these two types of trials are synthesized separately in order to clearly distinguish the effect of SSE in each experimental design.

### 2.3. Study Selection

Two authors, J.M.F.-G. and N.M.-P., screened the titles. We checked the titles and abstracts of the identified research for eligibility, independently reviewed the full text of the possibly eligible studies, chose the works that matched the inclusion criteria, and compared the results to achieve agreement. If eligibility was unclear, a third author (J.C.-V.) was consulted, and a decision was reached.

### 2.4. Data Extaction

Two researchers extracted data from the included studies’ original papers, which were then examined by a third investigator. Data extraction was carried out with the assistance of a bibliographic manager. It included the author’s names, year of publication, sample size, participant characteristics, SSE intervention, SSE combined with other training methods, control characteristics, measures and tests, completion rate, average attendance, dropouts with reasons, and main findings.

### 2.5. Quality Appraisal

The methodological quality of the selected RCT and NRCT was directly obtained from the Physiotherapy Evidence Database (PEDro), a validated tool specifically designed to assess the methodological quality of clinical trials, focusing on key aspects such as randomization, blinding and data integrity [49]. Studies were categorized as excellent (score of 9–10), good (6–8), fair (4–5), or of poor quality (≤3) [50].

## 3. Results

### 3.1. Design and Samples

**Figure 1:**
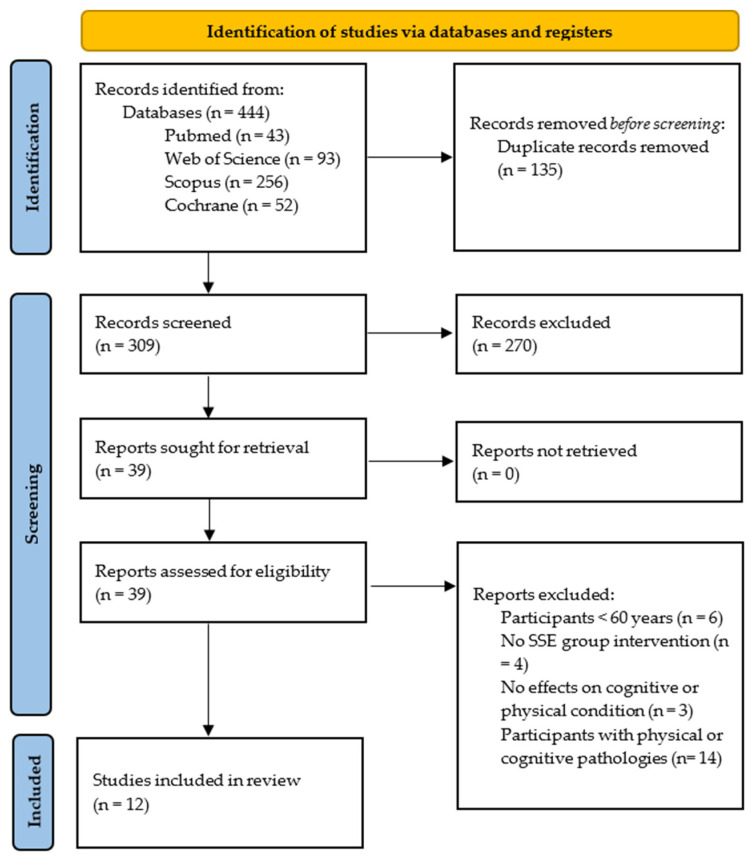
PRISMA flow chart illustrates the exclusion criteria and study selection.

Out of the initial pool of 444 records, 39 were selected for a full-text assessment, and initially 12 studies were chosen for the review. Following a citation search, no additional records were identified that met the inclusion criteria, resulting in a total of 12 studies finally included in the analysis (Figure 1). All research was published between 2006 and 2023.

The total sample size from all trials was 577 participants. In the NRCTs, the sample sizes of the intervention groups examined ranged from 20 to 44 participants, and in the RCTs from 10 to 32 participants. For the NRCTs, the sample size ranged from 2 to 18 men and 9 to 26 women, and for the RCTs from 0 to 14 men and 7 to 25 women. For both, the age range was 60 to 80 years. The mean height in the NRCTs was between 148.5 and 162 cm, and in the RCTs between 153 and 160 cm. Average body weight ranged from 52.5 to 70.8 kg in the NRCTs and from 55.6 to 67.5 kg in the RCTs.

The characteristics of the participant populations were homogeneous in terms of health status, as they were healthy at the time of the trials. For the NRCTs, variables of physical fitness, cognitive function and depressive symptoms were measured. For the RCTs, physical fitness variables, fear and fear of falling, heart rate variability, body fat and biomarkers were measured. Detailed information on the characteristics of the included trials is shown in Table 1.

### 3.2. Quality Appraisal

Table 2 shows the scores obtained for the assessment of methodological quality using the PEDro scale [49]. In this regard, two of the reviewed studies had a fair methodological quality [53,54], and ten were good [39,42,45,51,52,55,56,57,58,59]. A detailed description of the quality analysis was also included (see Table 2).

None of the research blinded the therapist, three blinded participants [39,45,59] and three blinded assessors measuring at least one key outcome [45,53,54]. In all trials examined, results were given for all participants who received or were assigned to the control group; when data for the control group could not be obtained, data for at least one major outcome were assessed using ‘intention to treat’. Furthermore, all studies included point and variability measurements for at least one major outcome.

### 3.3. Dropouts and Reasons

A total of 32 dropouts were observed in 3 of the 6 NRCTs that reported dropouts (see Table 3) [51,52,54]. Similarly, a total of 63 dropouts were reported in 5 of the 6 RCTs [39,55,56,58,59]. The main reasons for dropping out were similar in the NRCTs and RCTs, including medical or clinical reasons (back pain, knee pain, illness, hospitalization), discontinuation of the intervention and/or personal reasons, and failure to reach the minimum participation rate required for each study. None of the trials included in the review reported dropouts due to adverse effects of SSE.

### 3.4. General Characteristics of the Interventions

The main characteristics of the SSE interventions and strategies for each group are shown in Table 3. Regarding the control groups, two RCTs [57,58] and four NRCTs [42,51,52,53] maintained their activities of daily living [42,51,52,53,57,58].

Two RCTs [57,58] and three NRCTs [42,52,53] evaluated only one group receiving SSE training and a control group [42,52,53,57,58], although one of them (RCT) combined SSE training with pursed-lip breathing [57]. Four RCTs compared SSE training with other approaches, including walking [39], lower limb strength [55], balance [55,56] and Tai Chi Chuan [59]. Two NRCTs including lower limb strength, balance, flexibility, and aerobic endurance [51,54]. In addition, three NRCTs evaluated SSE combined with other training approaches to groups that incorporated SSE plus strength training [51], aerobic activity [45], and increasing 1000 steps per day [54]. No RCT evaluated SSE combined with other training approaches in groups incorporating SSE.

Sessions in the SSE-only and control group trials lasted between 30 and 70 min in the RCTS and between 30 and 60 min in the NRCTs, both including warm-up and cool-down periods. The intervention was delivered two to three times a week, and the program lasted between 12 weeks in the RCTs and 16 to 24 weeks in the NRCTs, with a frequency of one to three times a week. In trials comparing SSE with other exercise modalities, session durations ranged from 30 to 80 min in RCTs, with a weekly frequency from 1 to 3 times per week and a total protocol duration of 4 to 24 weeks. In the NRCTs, session durations ranged from 40 to 90 min, with a weekly frequency of 1 to 3 times per week and a total protocol duration of 9 to 16 weeks. In research that combined SSE with other training modalities, total session duration ranged from 40 to 90 min, weekly frequency from 1 to 3 times per week, and total treatment duration from 9 to 16 weeks for NRCTs. Three NRCTs [45,51,52] used subjective descriptors to describe training intensity, ranging from “mild” to “vigorous”.

The total duration of the interventions in the studies classified as NRCTs [42,45,51,52,53,54] ranged from 9 to 24 weeks. For example, Shigematsu et al. [42] used a 24-week intervention, while Jindo et al. [54] used a 9-week program. In contrast, studies with an RCT design [39,55,56,57,58,59] presented a more consistent intervention duration, with the majority being 12-week interventions, although some studies applied shorter durations, such as Sadeghian et al. [59] at 8 weeks and Bhanusali et al. [56], who implemented a 4-week intervention.

All the RCTs and NRCTs analysed used pre- and post-intervention assessments.

### 3.5. Main Outcomes

The primary findings of each study, as well as the measurement tests utilized for each variable, are presented in the following subsections in Table 4.

#### 3.5.1. Physical Function, Balance and Fear of Falling

Three studies that compared SSE-only to controls showed significant increases in agility, lower body strength, gait speed, flexibility and balance, two were NRCT but only compared within groups [42,53], and one was RCT comparing within and between groups [58]. In terms of NRCTs, Shigematsu et al. [42] used the Stand up from lying position test to assess agility and found significant improvements in the SSE group (*p* < 0.01). Pereira et al. [53] also found a significant increase in the Time up and go variable (*p* = 0.04). Shigematsu et al. [42] also found significant improvements (*p* < 0.05) in the lower limbs strength, walking speed and flexibility using the chair stand in 30 s, walking around two cones and sit and reach assessment tests. In terms of balance, Shigematsu et al. [42] and Pereira et al. [53] investigated the effects of their therapies using the single leg balance with eyes closed test, but only the first two experiments showed significant benefits (*p* < 0.05). Additionally, Pereira et al. [53] used the Berg Balance Scale, but no improvements were noted.

In the RCT, Cha et al. [58] found significant improvements in lower limb strength when comparing the SSE group with the control group on the Chair Stand in 30 s test (*p* < 0.01). Similarly, they found significant differences in balance within the SSE group when performing the single leg balance with eyes closed test.

Six trials reported mixed results when comparing the SSE group with other exercise programs, four of which were RCTs [39,55,56,59] and two of which were NRCTs [51,54].

In the RCTs, significant differences were found for agility [39,55,56,59], lower body strength [39,55,59], reaction time [39], walking speed [55], flexibility [55,59], balance [39,55,56,59], and fear of falling [56,59], but it is important to note that the study by Shigematsu et al. [55] only made within-group comparisons. In terms of agility, Shigematsu et al. [39,55] used the standing up from lying posture in two training protocols, the first comparing the SSE group with walking once a week for 12 weeks [39] and the second comparing the SSE group with traditional strength and balance training [55]; all groups improved in agility. Both studies also used the walking around two cones test and the stepping with both feet test; in the walking protocol, the SSE group showed greater improvements in both tests (*p* = 0.03; *p* = 0.04) [39]. However, in the strength and balance protocol, both groups showed significant improvements in agility (*p* = 0.003) [55]. Two pieces of research used the time up and go test, with inconsistent results. Bhanusali et al. [56] found that balance training produced better results in the SSE group (*p* = 0.001), but Sadeghian et al. [59] found significant gains in agility in the Tai Chi group (*p* = 0.032).

For the NRCTs, Teixeira et al. [51] observed significant changes in agility only in the combined SSE and basic exercise group (*p* = 0.002), but only within-group comparisons were made.

Three RCTs showed significant changes in limb strength and power. Shigematsu et al. [39,55] and Sadeghian et al. [59] used the chair stand 30 s test to assess lower limb strength and power, with Shigematsu et al. finding significant within-group improvements in both (*p* = 0.01). Sadeghian et al. also found improvements in both groups but found better results when comparing the Tai Chi group to the SSE group (*p* = 0.001). They also used the arm curl test, which showed significant benefits in this group for the upper extremities (*p* = 0.001). Shigematsu et al. also studied leg extension strength and found that it produced better results in SSE than walking training (*p* = 0.03) [39], as well as favourable results in both groups in the strength and balance protocol (*p* = 0.003) [55].

Only one NRCT showed significant changes in lower limb strength. Jindo et al. [54] showed that the SSE without goal setting group showed significant within group improvements in the five repetition sit-to-stand test by reducing the test time (*p* = 0.007).

Reaction time was observed in one RCT [39] and one NRCT [54]. Shigematsu et al. [39], used two tests to examine reaction time: simple reaction time and choice reaction time, with the SSE group showing significant improvements compared to the walking group (*p* < 0.001).

Jindo et al. [54] found within-group improvements in the SSE with goal setting group and the SSE without goal setting group for the choice reaction rime test (*p* = 0.03).

Walking speed was assessed in one RCT [55] and one NRCT [54]. Shigematsu et al. [55] assessed walking speed using the walking around two cones test and the ten meter walk and found improvements in the SSE group and the walking group (*p* = 0.003). Jindo et al. [54], used the walking ability test and found improvements in both groups (*p* = 0.024), but there was no comparison between groups.

Two RCTs assessed flexibility [55,59]. Shigematsu et al. [55] measured flexibility using sit and reach and found gains within groups but no differences between groups (*p* = 0.003). Sadeghian et al. [59] tested this using back scratch and found that the Tai Chi group performed better than the SSE group (*p* = 0.000).

Three RCTs [39,56,59] and one NRCT [51] investigated balance. The RCTs showed significant improvements in the SSE group compared to walking [39], balance training [56] and Tai Chi groups [59]. These results were based on forward tandem gait (*p* = 0.00) [39], backward tandem gait (*p* = 0.03) [39], and the Berg balance scale (*p* < 0.001) [56]. Sadeghian et al. [59], found that the Tai Chi group performed better on all measures used (one-legged balance with eyes closed (*p* = 0.002), one-legged balance with eyes open (*p* = 0.037), and functional reach (*p* = 0.016)). Shigematsu et al. [39] found increases in functional reach in both groups (*p* = 0.01).

For the NRCT, Teixeira et al. [51] found within group improvements in the SSE on the Time up and go test (*p* < 0.007) and in the alternative group on the Berg balance scale (*p* < 0.04).

Two RCTs used the Perceived Health Status measure to assess health status, with Shigematsu et al. [39] reporting higher scores in the SSE group compared to the walking group (*p* = 0.02). Bhanusali et al. [56] found that the SSE group performed better than the balance training group on the Fall efficacy scale (*p* < 0.001) and fear of falling (*p* < 0.001). Sadeghian et al. [59] found significant changes in the Tai Chi group (*p* < 0.001) compared to the SSE group on the Fall efficacy scale, which was not included in the health status.

Finally, only two NRCTs looked at SSE in combination with other training approaches for groups that integrated SSE with other types of training. Teixeira et al. [51] found that SSE combined with basic physical exercise improved within-group agility. Chang et al. [45] used the Time up and go to assess agility and found significant within-group improvements in all groups (*p* < 0.05, *p* < 0.05, *p* < 0.01); particularly, the general aerobic exercise +SSE and general aerobic exercise + ball game groups showed significant improvement compared to general aerobic exercise alone (*p* < 0.01). Chang et al. [45], used the Chair stands in 30 s test to assess strength and found significant gains in within group comparisons (*p* < 0.05). Chang et al. [45] also used the Single leg balance with eyes closed test to assess balance and found significant improvements in all groups (*p* < 0.05). Regarding gait, Teixeira et al. [51] found significant within group reductions in walking test time for the basic exercise group (*p* < 0.02), but not for any of the approaches including SSE, nor did they make any between-group comparisons.

#### 3.5.2. Cognitive Function

Only one RCT comparing SSE-only and control group interventions showed significant improvements in cognitive function scores in the SSE group between pre- and post-intervention [52], although no between-group comparisons were made. Teixeira et al. [52] found significant positive group by time interactions for general cognitive status (*p* = 0.002), concentrated attention, speed of response, performing simple task (*p* = 0.04), and executive function (*p* = 0.02) scores for the SSE group after 16 weeks of training three times a week, whereas the control group showed a significant decrease in general cognitive status and simple task performance, indicating a worse state in these functions.

There were no studies that compared cognitive function between SSE and other training programs, or between SSE groups combined with other training methods and groups combining SSE with other training.

#### 3.5.3. BDNF, IGF-1, and Vascular Health

Two RCTs compared the SSE-only group with the control group and found significant improvements in the SSE group between pre- and post-intervention within-group scores for blood BDNF and IGF-1 levels [58] and vascular health [57]. Cha et al. [58] found that 12 weeks of twice-weekly SSE training resulted in significant within-group improvements in BDNF expression levels in both the SSE group (*p* < 0.01) and the control group. There were no significant changes in blood BDNF or IGF-1 expression levels when compared between groups. Sawasdee et al. [57] examined the heart rate variability domain three times per week during a 12-week program of SSE training (mixed with Pursed-lip breathing), but found no changes within or between groups.

There were no RCTs or NRCTs that compared cognitive function between SSE and other training programs, or between SSE groups combined with other training methods and groups combining SSE with other training.

#### 3.5.4. Body Composition

Only one reviewed RCT comparing the SSE training group with the control group also measured body composition. Sawasdee et al. [57] measured body fat using skinfolds in the biceps, triceps, subscapularis, and supra-iliac muscles before and after 12 weeks of training three times a week. The SSE group showed a significant reduction in fat in all skinfolds (*p* < 0.05). Significant differences in the SSE group compared to the control group were found in the triceps, subscapular, and supra-iliac skin folds (*p* < 0.05).

#### 3.5.5. Depressive Symptoms

Of all the studies, only one NRCT looked at depressive symptoms. Pereira et al. [53] found that, after 16 weeks of twice-weekly SSE training, the intervention group showed no significant improvement in questionnaire scores, whereas the control group showed a significant increase in the questionnaire scores (*p* = 0.03), indicating that depressive symptoms increased in people who did not receive SSE training, but no comparisons were made between the two groups.

## 4. Discussion

This systematic review aimed to provide a thorough overview of the effects of using SSE on the physical-cognitive functioning of older adults. In addition to their effects on cognitive and physical function, the treatments were assessed for their effects on biomarkers, body composition and mental health. RCTs and NRCTs that evaluated the effectiveness of interventions based on the use of SSE were included in order to ensure the accuracy and relevance of the results. RCTs are considered the gold standard because of their ability to minimize bias through randomization, providing robust evidence on causal effects [60].

On the other hand, NRCTs, although at greater risk of bias, provide a more representative view of real-life scenarios, allowing conclusions that are more applicable to diverse populations and everyday settings [61]. This strengthens the external validity of the findings by reflecting the effectiveness of SSE in less controlled situations, which may be particularly important for interventions targeting older adults with different clinical or social characteristics.

In addition, the inclusion of studies with different methodological designs broadens the range of outcomes observed [60] allowing us to analyze not only the effectiveness of SSE under optimal control conditions, but also its feasibility and adherence in more realistic settings. This comprehensive approach facilitates the identification of potential barriers and facilitators to the implementation of SSE in older populations, a crucial aspect for translating these findings into clinical and community practice [62].

Another critical element to consider, in addition to methodological diversity, is the duration of interventions, as differences in the duration of exposure may significantly influence the observed outcomes. The duration of the interventions varies considerably between NRCT and RCT studies, with NRCTs ranging from 9 to 24 weeks and RCTs generally shorter and more uniform, mostly for 12 weeks, with some exceptions in the case of very short interventions, such as the study by Bhanusali et al. [56] with 4 weeks. Longer research, such as the 24-week NRCT by Shigematsu et al. [42], may allow for a deeper physiological and cognitive adaptation to SSE, which could positively influence the stability of the benefits obtained. In contrast, shorter interventions may limit the observed benefits, as effects on cognitive and physical function generally require longer exposure to manifest more consistently (at least for more than 12 weeks) [63,64]. This variability in duration raises the need to consider duration as a potential confounding factor when comparing results between studies and suggests that future research should standardize or explore different intervention periods to determine the optimal timing of SSE intervention in older adults.

### 4.1. Physical Function Outcomes

There were different characteristics in the physical functioning outcomes. First, when comparing the results of two NRCTs [42,53] and one RCT [58] that investigated the effects of SSE compared to control group, significant increases in agility, lower body strength and power, gait speed, flexibility and balance were found in this protocol [42,53,58]. These findings suggest that SSE is a useful form of training for healthy older adults to improve these fitness characteristics in those who have not previously exercised.

Four RCTs were identified in the review of articles comparing different training modalities with the SSE program. For agility, standard strength training and gait were examined, and both showed improvements comparable to those of SSE [39,55]. In comparison, SSE performed worse than Tai Chi [59] but better than a balancing protocol [56]. These differences may be due to the fact that Tai Chi involves a combination of postural control, directional change and continuous postural adjustment, unlike SSE, which follows a repetitive and predictable pattern. However, when strength and power were measured, SSE was found to produce more improvements than Tai Chi [59] and less than the walking protocol [39]. When it came to reaction time and balance, SSE outperformed the walking protocol [39] and, when it came to combined strength and balance training [55], Tai Chi also outperformed SSE in these areas, along with flexibility [59]. According to these findings, Tai Chi is more likely than SSE to promote physical fitness in healthy older adults. On the other hand, SSE was found to be more beneficial than walking and resulted in significant gains that were comparable to standard strength training.

Two NRCTs compared the effects of SSE in combination with other training with other types of training. These studies found that SSE with the aerobic protocol improved agility, lower limb strength and balance [45,51]. These results suggest that programs aimed at improving motor, aerobics, strength and balance skills may include or combine the use of SSE.

### 4.2. Cognitive Function

Only the NRCT by Teixeira et al. [52] found significant interactions with several aspects of cognitive function, including general cognitive status, focused attention, speed of response, performing simple tasks, and executive function. These results demonstrate that SSE has a significant positive effect on cognitive function. These gains were observed after 16 weeks of SSE training three times a week. It is important to note that the differences between the SSE group and the control group were not reported, which makes it difficult for us to assess the effects of SSE compared to the control group.

Several factors could explain these cognitive improvements. First, the aerobic nature of SSE may enhance synaptic plasticity by activating the central nervous system through improved neuronal communication efficiency [65,66,67]. Similarly, the improvement in executive function found by Teixeira et al. [52] may be due to the increased cognitive load associated with the coordination and planning required to perform SSE exercises. This demand may have strengthened the individuals’ executive abilities, as evidenced by the observed improvements. Furthermore, these data suggest that 12 to 16 weeks of therapy may be sufficient to produce improvements in cognitive performance.

There are few studies that explicitly compare cognitive function between SSE and other training programs, let alone those that combine SSE with other training approaches. This limitation prevents a comprehensive assessment of the relative effectiveness of SSE compared with other therapies. The lack of comparable research also makes it difficult to determine whether the observed effects are specific to SSE, or whether they can be replicated or even improved by combining SSE with other types of training.

### 4.3. BDNF, IGF-1, and Vascular Health

SSE training may have a good effect on certain specific biomarkers and vascular health, but significant benefits are not always seen when compared to controls. Two RCTs investigated the effects of an SSE program on biomarkers and vascular health. Cha et al. [58] found significant within-group increases in blood BDNF levels after 12 weeks of SSE training, but there were no significant differences in BDNF and IGF-1 levels between the SSE and control groups. This suggest that, while SSE may alter these biomarkers, it is not always more effective than other daily activities performed by the control group. Sawasdee et al. [57] examined heart rate variability during a 12-week program of SSE combined with pursed-lip breathing and found no significant differences within or between groups. This may mean that the combination of SSE and breathing, or the duration of the program, was insufficient to produce meaningful changes in hearth rate variability.

The observed results could be due to a number of variables. First, the frequency and duration of the SSE program may not have been sufficient to produce substantial changes in some biomarkers when compared to the control group. Individual differences in response to exercise could also have influenced the results, as biological variability can affect how participants respond to the intervention [68]. Another important factor to consider is the composition of the control group in the study by Cha et al. [58]. If the control group regularly participated in activities that could alter BDNF and IGF-1 levels (such as maintaining an active lifestyle, engaging in cognitive activities, practicing mindfulness, or playing chess) [69,70], the ability to detect significant changes between groups may have been reduced. Therefore, future research needs to carefully analyze the characteristics of the control group to ensure that comparisons are valid and adequately reflect the specific effects of SSE.

### 4.4. Body Composition

Body composition analysis in SSE training trials shows good results. The RCT by Sawasdee et al. [57] compared an SSE intervention group with a control group by measuring body fat in the biceps, triceps, subscapular and supra-iliac skinfolds before and after 12 weeks of training three times a week. The results showed a significant reduction in fat in all skinfolds assessed in the intervention group, as well as significant differences in favor of the training group in the triceps, subscapular, and supra-iliac skinfolds.

These significant reductions in body fat may be due to SSE, a type of aerobic exercise that, when performed regularly, can increase calorie expenditure and promote fat oxidation [71]. The design of SSE, which involves rapid and synchronized movements, may increase the intensity of exercise, resulting in greater body fat loss [72]. However, it should be noted that the study by Sawasdee et al. [57] is the only one of the research projects reviewed that assessed body composition. The lack of other related studies limits the generalizability of the data and emphasizes the need for further research into the effects of SSE on body composition. In addition, the study design and method of measuring body fat, such as via skinfolds, may influence the results. Skinfolds are a standard but indirect marker of body fat that can vary depending on the measurement technique [73,74]. Similarly, the individuals’ energy expenditure during the intervention period was not assessed.

### 4.5. Depressive Syntoms, Subjective Vitality and Group Cohesion

The results of this comprehensive review indicate that SSE may have a positive impact on certain domains of mental health and social cohesion, while the benefits on depressive symptoms are inconclusive. The NRCT by Pereira et al. [53] assessed the effect of SSE on depressive symptoms using the Geriatric Depression Scale Short Form after 16 weeks of twice-weekly training. The intervention group did not show a significant improvement, but the control group did, suggesting that SSE may prevent a worsening of depressive symptoms.

Research shows that regular PA improves mental health by reducing depressive symptoms and increasing vitality and social cohesion [75]. SSE, as a structured form of exercise, may provide a good program to promote these beneficial outcomes. However, future research needs to consider parameters such as exercise intensity, frequency and modality, as well as individual variability in response to training, in order to improve SSE programs aimed at promoting mental health.

This review provides useful and detailed information on the effects of SSE training on cognitive and physical function in healthy older people. One of the main strengths is the identification of studies that used square step exercises and were able to demonstrate the minimum and maximum intervention times. In addition, the study included an experimental group and a control group, which allowed us to validate changes in the variables.

However, the review had some limitations. Firstly, the number of research projects included was limited and the training methods used varied widely. As a result, we recommend that future research focuses on developing and evaluating interventions that combine SSE with other exercise modalities, as well as investigating a wider range of physical, psychological and social outcomes. In addition, the requirement that articles be written in English may have led to the exclusion of important studies published in other languages. Another limitation is that we did not perform a meta-analysis of the selected research due to the heterogeneity of the duration, sample, instruments used and research objectives of the selected studies, and therefore cannot recommend a minimum or maximum duration for interventions to achieve their benefits. In addition, not all trials reported the same descriptive data, such as weight and height. Another limitation is the need to cheek for changes and sex differences between experimental and control groups. Yet another is the lack of consistency in the intensity of the SSE interventions, which may have influenced the observed results. Only three NRCTs [45,51,52] reported exercise intensity, but this was not clearly specified, making it difficult to assess its direct effect on the benefits obtained. This suggests that some effects may be related to an overall increase in PA levels and not exclusively to the type of exercise performed [76]. Future research should include objective measures of intensity, such as heart rate or accelerometry, to distinguish the specific effects of SSE from those associated with a general increase in PA.

### 4.6. Practical Applications

SSE provides an accessible and adaptable tool for promoting physical and cognitive health in healthy older people. This type of exercise, which combines repetitive movements with the need for postural control and attention, is particularly useful in clinical and community settings where resources may be limited. The simplicity of SSE allows it to be delivered in both group and individual sessions, which promotes adherence through the social component and group motivation, benefiting both social cohesion and the emotional well-being of participants. In addition, the improvement in balance and coordination that SSE provides may directly contribute to the prevention of falls, an important goal in promoting quality of life in older adults.

The documented benefits suggest that SSE is a suitable alternative for those wishing to improve physical function without the need for complex equipment. Furthermore, SSE could be integrated into rehabilitation programs as a complement to other therapies, taking advantage of its ability to improve executive function and reduce the risk of falls. Therefore, SSE represents a low-cost and highly effective intervention that can be easily adapted to the specific needs of each individual or group, facilitating its inclusion in public health policies aimed at promoting healthy ageing.

### 4.7. Future Proposals

Despite the observed benefits, further research on SSE is needed to establish its comparative effectiveness and applicability in different populations of older adults. Future research should focus on randomized clinical trials that examine the effect of SSE in populations with pre-existing conditions, such as those with mobility limitations or mild cognitive impairment, to determine its therapeutic potential in a wider range of settings.

Another valuable line of research is to standardize the duration and intensity of programs, as the studies reviewed showed considerable variability in these aspects. Identifying the optimal duration of interventions is crucial to maximizing benefits and facilitating the recommendation of SSE in clinical guidelines. In addition, the inclusion of objective measures, such as the use of accelerometry to monitor exercise intensity, could improve the accuracy of assessing the effects of SSE and its underlying mechanisms.

## 5. Conclusions

The benefits of Square Stepping Exercise on physical function include increased agility, lower body strength, gait speed, flexibility, and balance. Although there are conflicting data on the benefits of SSE on cognitive performance, it may improve general wellbeing and prevent worsening of depressive symptoms. There were some improvements in BDNF, but no discernible changes in IGF-1. The results for BDNF and IGF-1 are mixed. While there are no discernible changes in vascular health with SSE, a significant reduction in body fat has been shown. Overall, SSE offers significant gains in physical function and body composition, with possible psychological benefits and variable effects on biomarkers and vascular health.

## Figures and Tables

**Table 1:** Characteristics of participants and measures.

Authors (Year)	Study Desing	Sample	Group	n	Sex (Men/Women)	Age Criteria (Years)	Age (Years)	Height (cm)	Weight (Kg)	Measures (Test)
Shigematsu et al., (2006) [42]	NRCT	Initial/final: 52/52	EG	26	6/20	60–80	67.5 ± 4.9	148.5 ± 4.3	53.2 ± 7.0	Agility (SULP); Leg Power (CH10S); Locomotion Speed (WTC); Flexibility (SR); Balance (SLBEC)
			CG	26	-		68.3 ± 6.3	151.4 ± 6.3	52.5 ± 9.3	
Teixeira et al., (2013) [51]	NRCT	Initial/final: 106/86	EG	21	12/9	>60	68.2 ± 8.4	161 ± 10	70.8 ± 15.6	Agility (s); Strength (repetitions); Coordination (s); Walking (s); Flexibility (cm); Balance (BBS, TUG, TUGS)
			BE	20	4/16		66.9 ± 5.3	162 ± 10	70.2 ± 12.1	
			SSE/BE	25	6/19		67.1 ± 5.8	156 ± 10	64.2 ± 11.3	
			CG	20	2/18		67.9 ± 6.7	156 ± 10	68.5 ± 15.2	
Teixeira et al., (2013) [52]	NRCT	Initial/final: 50/41	EG	21	12/9	>60	68.2 ± 8.4	-	-	General cognitive status (MMSE); Short-term memory and ability to mentally manipulate information (DSTF, DSTB); Concentrated attention, speed of response, performing simple task (TPS, TPQ); Executive function (MCST)
			CG	20	2/18		67.9 ± 2.9			
Pereira et al., (2014) [53]	NRCT	Initial/final: 32/32	EG	15	-	>60	73.24 ± 9.59	-	-	Depressive symptoms (GDS-15); Functional mobility (TUG); Balance (BBS)
			CG	17	-		79.4 ± 8.01			
Jindo et al., (2016) [54]	NRCT	Initial/final: 35/32	GS	19	4/15	>60	68.9 ± 3.3	-	-	Reaction speed (CRT); Muscle strength (5STS); Mobility (TUG); Balance (SLBEO); Walking ability (5HW); Endurance (6MW); PA
			WGS	13	2/11		69.9 ± 4.2	-	-	
Chang et al., (2017) [45]	NRCT	Initial/final: 102/102	GAE + SSE	28	8/20	>65	74.3 ± 4.7	-	-	Aerobic endurance (SIP); Mobility (TUG); Leg Strength (CH30S); Flexibility (SR); Reaction time (BGRT); Static balance (SLBEC)
			GAE + BG	30	12/18		75.5 ± 4.0	-	-	
			GAE	44	18/26		78.4 ± 5.2	-	-	
Shigematsu et al., (2008) [39]	RCT	Initial/final: 68/63	EG	32	14/18	65–74	68.6 ± 2.4	153.3 ± 10.0	59.3 ± 11.2	Agility (SULP, SBF, WTC); Leg Strength Power (CH30S, LEP); Reaction (SRT, CRT); Balance (SLBEC, FR, FTW, BTW); FOF; PDE; PHS
			WG	31	6/25		69.5 ± 2.9	154.4 ± 2.9	55.6 ± 7.6	
Shigematsu et al., (2008) [55]	RCT	Initial/final: 39/39	EG	20	10/10	65–74	68.8 ± 2.4	156.8 ± 9.6	58.4 ± 11.2	Agility (SULP, SBF); Walking Speed (WTC, 10MW); Leg Strength; Power (CH30S, LEP); Balance (SLBEC, FR); Flexibility (SR)
			SB	19	11/8		69.3 ± 3.2	158.5 ± 9.4	61.9 ± 11.5	
Bhanusali et al., (2016) [56]	RCT	Initial/final: 40/36	EG	18	-	>60	-	-	-	Balance (BBS, TUG, FES, PQ)
			BT	18	-					
Sawasdee et al., (2020) [57]	RCT	Initial/final: 43/43	EG	22	1/22	>65	69.59 ± 4.35	154.6 ± 6.4	61.3 ± 8.9	Heart Rate Variability (SDNN, rMSSD, TP, VLF, LF, HF, LF/HF); Body fat (biceps, triceps, subscapular, supra-iliac)
			CG	21	4/21		68.95 ± 4.13	154.4 ± 8	62.1 ± 12.3	
Cha et al., (2022) [58]	RCT	Initial/final: 38/20	EG	10	3/7	>60	74.8 ± 6.76	158.1 ± 7.7	58.1 ± 7.2	Leg Strength Power (CH30S); Balance (SLBEC); BDNF; IGF-1
			CG	10	3/7		72.5 ± 6.52	160.4 ± 6.3	59.8 ± 8.22	
Sadeghian et al., (2023) [59]	RCT	Initial/final: 74/36	EG	18	0/18	>60	65.06 ± 4.09	155 ± 0.07	67.5 ± 9.32	Functional fitness (SLBEC SLBEO, TUG, FR, CH30S, AC, BS); Fear of Falling (FES)
			TG	18	0/18		65.33 ± 3.57	156 ± 0.05	67.2 ± 11.3	

5HW: 5-m habitual walk, 5STS: Five-repetition sit-to-stand, 6MW: 6-min walk, 10MW: ten meters walk, AC: Arm curl test, BBS: Berg Balance Scale, BDNF: Brain-derived neurotrophic factor, BE: Basic physical exercise, BG: Ball game, BGRT: Bar-gripping reaction time test, BS: Back scratch test, BT: Balance training, BTW: Backward tandem walking, CG: control group, CH10S: Chair stands in 10 s, CH30S: Chair stands in 30 s, cm: centimeters, CRT: Choice reaction time, DSTF: Digit Span test Forward, DSTB: Digit Span test Backward, EG: experimental group, FES: Fall efficacy scale, FOF: Fear of falling, FR: Functional reach, FTW: Forward tandem walking, GAE: General aerobic exercise, GDS-15: Geriatric depression scale short form, GS: goal-setting group, HF: High frequency, IGF-1: insulin-like growth factor-1, LEP: Leg extension power, LF: Low frequency, LF/HF: LF and HF ratio, MCST: Modified Card Sorting Test, MMSE: Mini-Mental State Examination, NRCT: non-randomized control trial, PA: Physical activity, PDE: Pleasure during exercise, PHS: Perceived health status, PQ: Tare of likelihood of fall, RCT: randomized control trial, rMSSD: Mean squares differences of successive normal-to-normal intervals, s: seconds, SB: Traditional strength and balance exercise training group, SBF: Stepping with both feet, SDNN: Standard deviation of all normal-to-normal intervals, SLBEC: Single-leg balance with eyed closed, SLBEO: Single-leg balance with eyed open, SIP: Stepping in a place, SR: Sit and Reach, SRT: Simple reaction time, SSE: Square-stepping exercise, SSE/SB: basic physical exercises and the sequences of SSE, SULP: Standing up from a lying position, TG: Tai Chi Chuan group, TP: Total power, TPS: Toulouse–Pierón speed, TPQ: Toulouse–Pierón quality, TUG: Time up and go (time), TUGS: Time up and go (steps), VLF: Very low frequency, WG: Walking group, WGS: Without goal-setting group, WTC: walking around two cones.

**Table 2:** Results of the methodological quality assessment of included studies.

Study	PEDro Item											Score	Quality
	1	2	3	4	5	6	7	8	9	10	11		
Shigematsu et al., (2006) [42]	1	0	1	1	0	0	0	1	1	1	1	6	Good
Shigematsu et al., (2008) [39]	1	1	1	1	1	0	0	1	1	1	1	8	Good
Shigematsu et al., (2008) [55]	1	1	1	1	0	0	0	1	1	1	1	7	Good
Teixeira et al., (2013) [51]	1	0	1	1	0	0	0	1	1	1	1	6	Good
Teixeira et al., (2013) [52]	1	0	1	1	0	0	0	1	1	1	1	6	Good
Pereira et al., (2014) [53]	1	0	0	1	0	0	1	0	1	1	1	5	Fair
Bhanusali et al., (2016) [56]	1	1	1	1	0	0	0	0	1	1	1	6	Good
Jindo et al., (2016) [54]	1	0	0	0	0	0	1	0	1	1	1	4	Fair
Chang et al., (2017) [45]	1	0	1	1	1	0	1	0	1	1	1	7	Good
Sawasdee et al., (2020) [57]	1	1	1	1	0	0	0	0	1	1	1	6	Good
Cha et al., (2022) [58]	1	1	1	1	0	0	0	1	1	1	1	7	Good
Sadeghian et al., (2023) [59]	1	1	1	1	1	0	0	1	1	1	1	8	Good

PEDro items: 1: Eligibility criteria, 2: Random allocation, 3: Concealed allocation, 4: Baseline comparability, 5: Blind subjects, 6: Blind therapist, 7: Blind assessors, 8: Adequate follow-up, 9: Intention-to-treat analysis, 10: Between groups comparisons, 11: Point estimates and variability.

**Table 3:** Characteristics of completion rate.

Authors (Year)	Study Design	Group	Intervention					Completion Rate (%)	Average Attendance (%)	Dropouts	Reasons
			Type of Exercise	Duration (Weeks)	Frequency (Weekly)	Intensity	Session Duration (min)				
Shigematsu et al., (2006) [42]	NRCT	EG	SSE	24	Once	-	60	100	92	-	-
		CG	Usual lifestyles		-		-				
Teixeira et al., (2013) [51]	NRCT	EG	SSE	16	Three times	Mild effort	40	89.6	-	20	Giving up participation, clinical instability, not agreeing to participate in post-intervention evaluations, >75% absence in training sessions
		BE	Aerobic endurance, flexibility, muscular resistance and balance			Auto reported	40	83			
		SSE/BE	SSE + BE				20 for each	85.4			
		CG	Usual lifestyles								
Teixeira et al., (2013) [52]	NRCT	EG	SSE	16	Three times	Mild effort	40	82	>75	9	Gave up in participating in the intervention, >25% absence, not agree evaluation protocol
		CG	Usual lifestyles		-	-	-		-		
Pereira et al., (2014) [53]	NRCT	EG	SSE	16	Twice	-	30	-	-	-	-
		CG	Usual lifestyles								
Jindo et al., (2016) [54]	NRCT	GS	SSE + increase 1000 steps/day	9	Once	-	90	91.42	95.9 (±8.5)	3	Not attend post-test measurement, not complete a session.
		WGS	SSE						94 (±10.7)		
Chang et al., (2017) [45]	NRCT	GAE + SSE	AA + SSE	12	Twice	Moderate to vigorous	60	100	-	-	-
		GAE + BG	AA + throwing ball								
		GAE	AA								
Shigematsu et al., (2008) [39]	RCT	EG	SSE	12	Twice	-	70	100	92 (±12.1)	5	Family issues, unwillingness to continue participation, knee pain.
		WG	Walking		Once		40	88.84	84.2 (±23.7)		
Shigematsu et al., (2008) [55]	RCT	EG	SSE	12	Three times	-	70	100	87	2	Back pain, time conflict
		SB	Strength and balance exercise (3 set × 10 repetitions)		Twice		20 for each		83		
Bhanusali et al., (2016) [56]	RCT	EG	SSE	4	Three times	-	30	-	-	4	Illness, travel, loss on interest
		BT	Circuit of static and dynamic balance								
Sawasdee et al., (2020) [57]	RCT	EG	PLB + SSE	12	Three times	-	30	100	-	-	-
		CG	Usual lifestyles								
Cha et al., (2022) [58]	RCT	EG	SSE	12	Twice	-	70	>80	At least 21 sessions	18	Participation < 80%, personal circumstances, eye disease, hospitalization.
		CG	Usual lifestyles								
Sadeghian et al., (2023) [59]	RCT	EG	SSE	8	Three times	-	60–80	100	-	34	Not meeting inclusion criteria, interfering medical conditions
		TG	TG								

AA: Aerobic activity, BE: Basic physical exercise, BG: Ball game, BT: Balance training, CG: control group, EG: experimental group, GAE: General aerobic exercise, GS: goal-setting group, NRCT: Non randomized clinical trial, PLB: Pursed-lip breathing, RCT: Randomized clinical trial SB: Traditional strength and balance exercise training group, SSE: Square-stepping exercise, SSE/SB: basic physical exercises and the sequences of SSE, TG: Tai Chi Chuan group, WG: Walking group, WGS: Without goal-setting group.

**Table 4:** Main findings.

Authors (Year)	Study Design	Group	Intragroup	Intergroup
Shigematsu et al., (2006) [42]	NRCT	EG	↑SULP, ↑CH10S, ↑WTC, ↑SR, ↑SLBEC.	-
		CG	-	
Teixeira et al., (2013) [51]	NRCT	EG	↑TUG, ↑TUGS	-
		BE	↓Walking, ↑BBS	
		SSE/BE	↑Agility	
		CG	↓BBS, ↓TUG	
Teixeira et al., (2013) [52]	NRCT	EG	↑MMSE, ↑TPQ, ↓MCST	-
		CG	↓MMSE, ↓TPQ, ↑MCST	
Pereira et al., (2014) [53]	NRCT	EG	↑TUG	-
		CG	↑GDS-15	
Jindo et al., (2016) [54]	NRCT	GS	↑5HW, ↑CRT, ↑6MW	-
		WGS	↑5HW, ↑CRT, ↑6MW, ↓5STS	
Chang et al., (2017) [45]	NRCT	GAE + SSE	↑SIP, ↑CH30S, ↑SLBEC, ↑TUG	GAE+ SSE and GAE + BG ↑TUG compared to GAE
		GAE + BG		
		GAE		
Shigematsu et al., (2008) [39]	RCT	EG	↑CH30S, ↑FR, ↑SULP	EG ↑LEP, ↑FTW, ↑BTW, ↑SBF, ↑WTC, ↑SRT, ↑CRT, ↑PHS compared to WG
		WG		
Shigematsu et al., (2008) [55]	RCT	EG	↑SLBEC, ↑CH30S ↑LEP, ↑SBF, ↑WTC, ↑10MW, ↑SR	-
		SB	↑CH30S ↑LEP, ↑SBF, ↑WTC, ↑10MW, ↑SR	
Bhanusali et al., (2016) [56]	RCT	EG	↑BBS, ↑TUG, ↑FES, ↑PQ	EG ↑BBS, ↑TUG, ↑FES, ↑PQ compared to BT
		BT	↑BBS, ↑TUG, ↑FES, ↑PQ	
Sawasdee et al., (2020) [57]	RCT	EG	↑biceps, triceps, subscapular, supra-iliac	EG ↑triceps, subscapular, supra-iliac compared to CG
		CG	-	
Cha et al., (2022) [58]	RCT	EG	↑CH30S, ↑SLBEC, ↑BDNF	EG ↑CH30S compared to CG
		CG	↑BDNF	
Sadeghian et al., (2023) [59]	RCT	EG	↑SLBEC, ↑SLBEO, ↑TUG, ↑FR, ↑CH30S, ↑AC, ↑BS, ↑FES	TG ↑SLBEC, ↑SLBEO, ↑TUG, ↑FR, ↑CH30S, ↑AC, ↑BS compared to EG
		TG		

5HW: 5-m habitual walk, 5STS: Five-repetition sit-to-stand, 6MW: 6-min walk, 10MW: ten meters walk, AC: Arm curl test, BBS: Berg Balance Scale, BDNF: Brain-derived neurotrophic factor, BE: Basic physical exercise, BG: Ball game, BS: Back scratch test, BT: Balance training, BTW: Backward tandem walking, CG: control group, CH10S: Chair stands in 10 s, CH30S: Chair stands in 30 s, cm: centimeters, CRT: Choice reaction time, EG: experimental group, FES: Fall efficacy scale, FR: Functional reach, FTW: Forward tandem walking, GAE: General aerobic exercise, GDS-15: Geriatric depression scale short form, GS: goal-setting group, LEP: Leg extension power, MCST: Modified Card Sorting Test, MMSE: Mini-Mental State Examination, NA: not available, NRCT: Non randomized clinical trial, PHS: Perceived health status, PQ: Tare of likelihood of fall, RCT: Randomized clinical trial, SB: Traditional strength and balance exercise training group, SBF: Stepping with both feet, SLBEC: Single-leg balance with eyed closed, SLBEO: Single-leg balance with eyed open, SIP: Stepping in a place, SR: Sit and Reach, SRT: Simple reaction time, SSE: Square-stepping exercise, SSE/SB: basic physical exercises and the sequences of SSE, SULP: Standing up from a lying position, TG: Tai Chi Chuan group, TPQ: Toulouse–Pierón quality, TUG: Time up and go (time), TUGS: Time up and go (steps), WG: Walking group, WGS: Without goal-setting group, WTC: walking around two cones, ↓: worsens, ↑: improves.

## Data Availability

Data are contained within the article.
